# Genome-Wide Identification and Analysis of *Catharanthus roseus* Receptor-like Kinase 1-like Proteins in Eggplant

**DOI:** 10.3390/plants12193379

**Published:** 2023-09-25

**Authors:** Wenpeng Ma, Juan Du, Xinlong Yu, Kai Chen, Yucheng Ming, Libo Jiang, Tong Chen, Dongchao Ji

**Affiliations:** 1 College of Agricultural Engineering and Food Science, Shandong University of Technology, Zibo 255049, China; 2 School of Life Sciences and Medicine, Shandong University of Technology, Zibo 255049, China; 3 Key Laboratory of Plant Resources, Institute of Botany, Innovative Academy of Seed Design, Chinese Academy of Sciences, Beijing 100093, China; 4 University of Chinese Academy of Sciences, Beijing 100049, China; 5 China National Botanical Garden, Beijing 100093, China

**Keywords:** *Catharanthus roseus* receptor-like kinase 1-like protein, eggplant, gene family, expression analysis

## Abstract

As an important member of the plant receptor-like kinases, *Catharanthus roseus* receptor-like kinase 1-like (CrRLK1L) plays vital roles in plant growth and development, as well as biotic and abiotic stress response. Numerous CrRLK1Ls have been identified and analyzed in various plant species, while our knowledge about eggplant (*Solanum melongena* L.) CrRLK1Ls is still scarce. Utilizing state-of-the-art genomic data, we conducted the first genome-wide identification and analysis of CrRLK1L proteins in eggplant. In this study, 32 CrRLK1L proteins were identified and analyzed in eggplant. A subsequent gene structure and protein domain analysis showed that the identified eggplant CrRLK1Ls possessed typical features of CrRLK1Ls. A subcellular localization prediction demonstrated that these proteins mostly localized on the plasma membrane. A collinearity analysis showed that some eggplant *CrRLK1L* genes had predicted intraspecies or interspecies evolutionary duplication events. Promoter analysis suggests that eggplant CrRLK1Ls may be involved in plant hormone signaling, host–pathogen interactions, and environmental responses. Based on transcriptomic gene expression analysis, it is indicated that eggplant *CrRLK1Ls* may be involved in the resistance response of eggplant to *Botrytis cinerea*. Together, these results will give us a theoretical foundation and guidance for elaborating the biological functions of CrRLK1Ls in eggplant growth, development, and resistance response.

## 1. Introduction

As the largest receptor subfamily in plants, receptor-like kinases (RLKs) play significant roles in plant growth and development, as well as stress and pathogen responses [1,2]. Initially identified in *Catharanthus roseus*, plant-specific CrRLK1L protein kinases have since been discovered in numerous plant species, garnering attention from researchers worldwide [2,3,4]. Traditionally, the typical domain structure of CrRLK1L consists of a malectin-like domain, transmembrane domain, and kinase domain [4]. The malectin-like domain was considered to be bound and received extracellular signals, while the kinase domain usually exerted phosphatase activity [2,5,6]. CrRLK1Ls were reported to be involved in a wide range of biological processes regulation, including cell expansion, hormone signaling, male–female gametophyte recognition, energy production, stress tolerance, and host–pathogen interactions [2,7,8,9,10,11,12].

FERONIA (FER), initially identified in an *Arabidopsis* female gametophytic mutant, stands as one of the most extensively investigated CrRLK1L proteins [4,13,14]. FER has been implicated in various biological pathways, the including abscisic acid (ABA), auxin, brassinosteroid (BR), ethylene, and jasmonic acid (JA) hormone responses, as well as the *Pseudomonas syringae* pv. tomato DC3000, *Fusarium oxysporum*, and *Golovinomyces* (*syn*. *Erysiphe*) *orontii* pathogen responses [7,15,16,17,18,19,20,21].

Additionally, the extracellular ligands of FER have progressively come to light. Rapid Alkalization Factors (RALFs), were identified to serve as the ligands, together with FER, to trigger different biological processes [22,23,24]. RALFs are widely distributed in plants. There are about 34 members in *Arabidopsis* [25]. Notably, RALF1 plays a crucial role in plant cell expansion. RALF1 interacts with the extracellular domain of FER, inhibiting proton transport and cell elongation through the phosphorylation of serine 899 of the plasma membrane H+–adenosine triphosphatase 2 (AHA2) [8]. Cell elongation in plants primarily relies on the expansion of vacuoles [26]. The extracellular proteins leucine-rich repeat extension 3/4/5 (LRX3/4/5) and RALF1 interact with FER, forming a functional module that collectively sense and transmit cell wall signals, inhibiting vacuole expansion and cell elongation [26]. Upon sensing the RALF1 signal, FER additionally orchestrates the recruitment and phosphorylation of the cytoplasmic receptor-like kinase RIPK (Resistance to *Pseudomonas syringae* pv. maculicola 1-induced protein kinase), culminating in the suppression of root elongation [27]. Furthermore, in response to the RALF1 signal, FER fosters mRNA’s translation and phosphorylation of ErbB3-binding protein 1 (EBP1). This prompts its nuclear accumulation and ensuing repression of the transcription of the RALF1-associated gene *calmodulin-like protein 38* (*CML38*), thereby establishing negative feedback for the RALF1 signal [28,29]. Under normal circumstances, FER would undergo both clathrin-dependent and clathrin-independent endocytosis. RALF1 enhances the clathrin-dependent endocytosis of FER, and blocking the clathrin-dependent endocytic pathway could slow down RALF1-mediated root growth inhibition [30]. In addition, another study has shown that FER, in association with RALF1, regulates the mRNA translation and local protein synthesis of the transcription factor RSL4 (root hair defective 6-like 4) to inhibit the transcription level of *RALF1*, and promotes root hair tip growth through the phosphorylation of eukaryotic translation initiation factor 4E1 (eIF4E1) [31].

Several other members of the *Arabidopsis* CrRLK1L family have been functionally characterized. Specifically, AtTHESEUS1 and AtHERCULES1 have been linked to cell elongation [32,33], while AtANXUR1/2 and AtBUPS1/2 (AtBUDDHA’S PAPER SEAL1/2) are involved in regulating pollen tube growth [12,34], and AtMEDOS1 (AtMDS1), AtMDS2, AtMDS3, and AtMDS4 are involved in the metal ion stress response [35].

As an important economic vegetable crop, eggplant (*Solanum melongena* L.), is widely grown and consumed worldwide. Eggplant genome sequencing and assembly has been completed in recent years [36,37]. A variety of CrRLK1L members have been identified and analyzed in *Arabidopsis* [38], rice [39], apple [40], strawberry [41], soybean [42], Citrus [43], cotton [44], pear [45], tobacco [46], potato [47] and tomato [48,49]. However, little is known about the CrRLK1L members in eggplant, and our knowledge about the function of these proteins is very limited. Therefore, the identification of eggplant CrRLK1Ls (SmCrRLK1Ls) is valuable for the study of this plant. In this study, with access to the state-of-the-art eggplant genome, a genome-wide systematic identification and an analysis of SmCrRLK1L genes and proteins using bioinformatics were performed. This research will provide us with new insights and clues to better understand the SmCrRLK1Ls family and lay the foundations for revealing the function of these proteins. This study also establishes a fundamental groundwork for the further exploration of SmCrRLK1L’s function in plant–microbe interactions.

## 2. Results

### 2.1. Identification of CrRLK1L Proteins in Eggplant

The simple HMM search tool from TBtools was used to identify the eggplant CrRLK1Ls. Pfam files (pfam: PF12819 and PF07714) were used as queries to search the eggplant databases in the SGN. After searching, the scores of the domain and sequence were acquired, respectively (Dataset S1). Taking the intersection of the domain and sequence score classifications, 32 *Cr*RLK1L protein candidates were identified in eggplant, named SmCrRLK1L1 (*Solanum melongena* L. CrRLK1L1) to SmCrRLK1L32 according to their location on the chromosomes (Figure S1, Table 1). Subsequent research revealed that the smallest and largest proteins were SmCrRLK1L29 and SmCrRLK1L8, which had 443 and 1084 amino acids, respectively, coinciding with their molecular weight (MW). At the same time, the theoretical isoelectric point (pI), instability index, aliphatic index, and grand average of hydropathicity (GRAVY) of these 32 SmCrRLK1L proteins were analyzed. The results are listed in Table 1. The pI ranged from 5.27 to 9.05, the instability index ranged from 27.96 to 46.93, the aliphatic index ranged from 79.51 to 97.1, and the GRAVY was −0.298 to 0.025. The instability index is a measure that predicts the stability of a protein in a test tube [50]. If the instability index is less than 40, the protein is predicted to be stable, while a value exceeding 40 suggests potential instability. SmCrRLK1L2 to 4, 10 to 13, 23, 28, and 29 were predicted as unstable proteins, and the rest were considered stable.

### 2.2. Phylogenetic Analysis of the SmCrRLK1Ls

**Figure 1:**
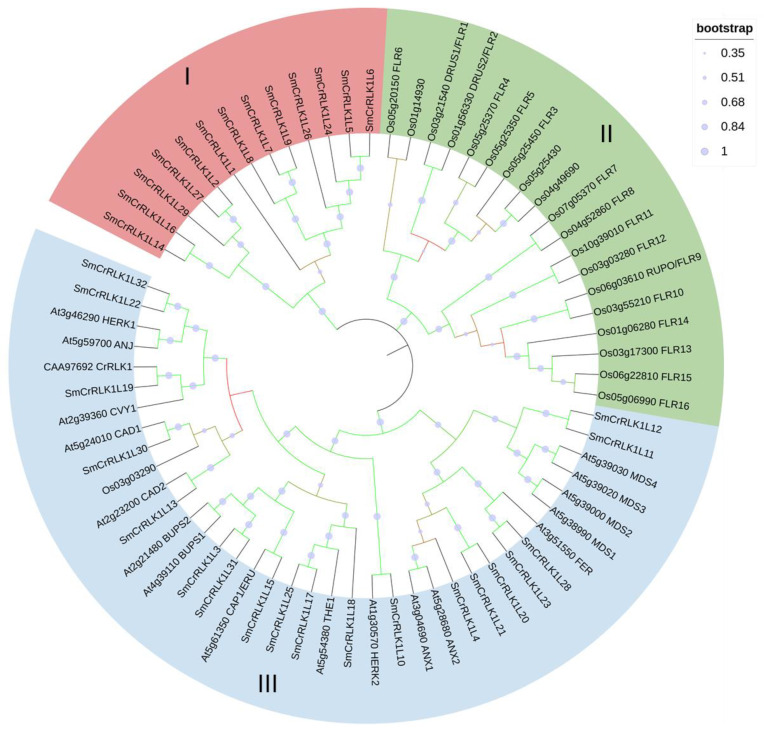
Phylogenetic analysis of CrRLK1Ls in eggplant, *Catharanthus roseus*, *Arabidopsis*, and rice. Different groups are represented by different colors. The bootstrap metadata display was indicated by symbol circles and color gradient (red to green: 0.352 to 1). I, II and III refer to the subfamilies divided by phylogenetic analysis.

To explore the relationship among *Catharanthus roseus,* eggplant, *Arabidopsis*, and rice CrRLK1Ls, a phylogenetic analysis was conducted using MEGA (Figure 1). As a result, the CrRLK1Ls could be divided into three subfamilies. As shown in Figure 1, subfamilies I, II, and III owned 13, 19, and 38 members, respectively. All *Arabidopsis* CrRLK1L members were distributed in subfamily III. The rice CrRLK1Ls had 19 members in subfamily II, and 1 member in subfamily III. The *Catharanthus roseus* CrRLK1 was in group III. The eggplant CrRLK1L proteins were mainly distributed in subfamilies I and III, suggesting that the evolutionary relationship between *Arabidopsis* and eggplant was much closer than that between *Arabidopsis* and rice. Meanwhile, subfamily I only had eggplant CrRLK1L proteins, indicating that these proteins might have newly unknown functions that need to be explored in the future.

### 2.3. Chromosome Distribution of SmCrRLK1Ls

**Figure 2:**
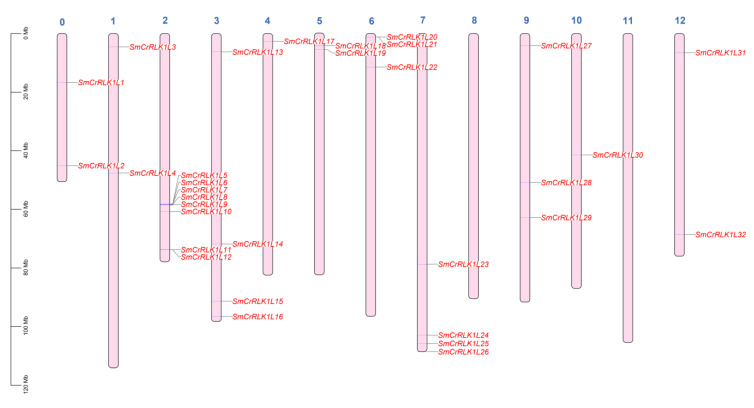
Chromosome location of *SmCrRLK1Ls*. Unit: Mb. Chromosomes 0 to 12 were shown as indicated; the length of the figure represents the corresponding chromosome length. The location of the *SmCrRLK1Ls* genes were labeled as red words from *SmCrRLK1L1* to *SmCrRLK1L32*.

To understand the relationships between *SmCrRLK1L* genes, their chromosomal distribution information was obtained from the SGN and analyzed. The results showed that the *SmCrRLK1L* genes were not equally dispersed across the chromosomes. As shown in Figure 2, all the *SmCrRLK1L* genes were distributed on chromosomes 1 to 7, 9, 10, 12, and 0, but not on chromosomes 8 and 11. Chromosome 2 owned 8 *SmCrRLK1L* genes, while the number of *SmCrRLK1L* genes in chromosomes 4 and 10 was one (Figure 2). *SmCrRLK1L1* and *2* were located on chromosome 0. Traditionally, the annotation of chromosome 0 is not clear and needs to be deeply analyzed in the future. Thus, the annotations of *SmCrRLK1L1* and *2* could be further optimized.

### 2.4. Protein Domain and Gene Structure Analysis of SmCrRLK1L

**Figure 3:**
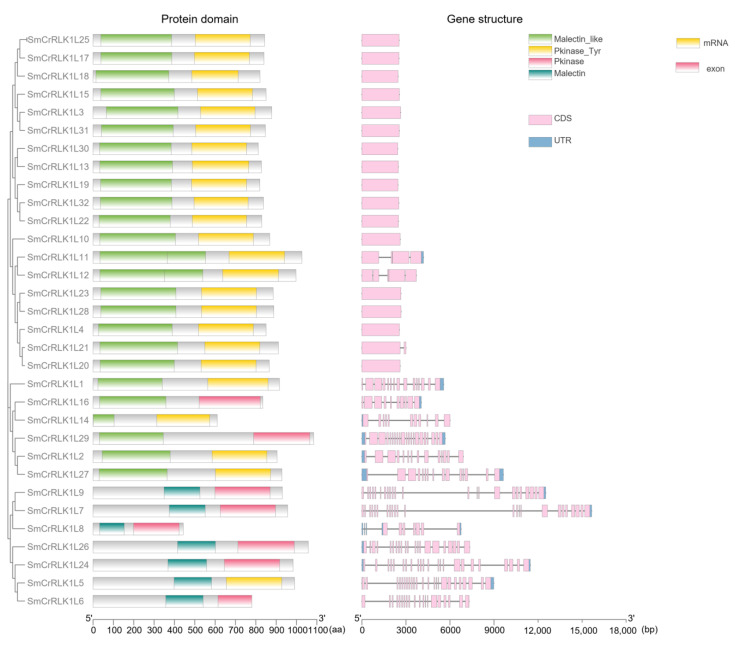
Protein domain and gene structure of SmCrRLK1Ls. The domains are marked in different colors. CDS: coding sequence; UTR: untranslated region.

The characteristic domain configuration of CrRLK1L typically consisted of a malectin-like domain, a transmembrane domain, and a kinase domain. In order to further confirm and analyze the SmCrRLK1L proteins, conserved protein domain and gene structure detection were performed. All the SmCrRLK1L sequences were submitted to PfamScan (https://www.ebi.ac.uk/Tools/pfa/pfamscan/) (accessed on 7 October 2022) to search for the conserved domains. As shown in Figure 3 and Dataset S3, the malectin, malectin_like, Pkinase, and Pkinase_Tyr domains were verified, which are the traditional domains of CrRLK1Ls. Gene structure analysis showed that 16 of the 32 *SmCrRLK1L* genes possessed continuous CDSs, which was another typical characteristic of the *CrRLK1L* gene family (Figure 3). These results further confirmed the correctness of the protein family we obtained.

### 2.5. Conserved Motifs and Subcellular Localization of SmCrRLK1L Prediction

**Figure 4:**
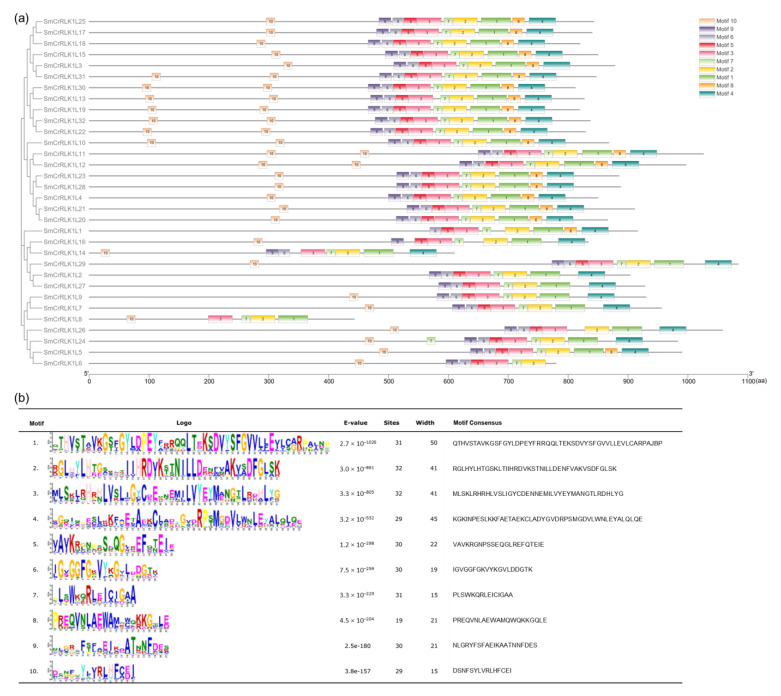
Conserved motif analysis of SmCrRLK1Ls. (**a**) Motif locations on SmCrRLK1L proteins. (**b**) The motif consensus and logo are listed. Sites: the number of sites contributing to the construction of the motif.

The conserved motifs were analyzed using MEME. As a result, 10 conserved motifs were acquired in total (Figure 4), and the amino acid numbers ranged from 15 to 50. As shown in Figure 4, motifs 2 and 3 were distributed across all 32 members, and motifs 1, 4, 5, 6, 7, 9, and 10 could be found in almost all 32 members. The common characteristic motifs between the SmCrRLK1Ls indicate that these proteins may have similar functions.

Previous studies have reported that most plant CrRLK1Ls are plasma membrane proteins [40,42,45,46,48]. In this study, we used CELLO to determine the localization of the SmCrRLK1Ls. The results were as follows: As shown in Table 2 and Dataset S4, most SmCrRLK1Ls were predicted to be membrane proteins, which was consistent with the previous report. Meanwhile, the signal peptide (SP) and transmembrane helix (TMH) prediction of the SmCrRLK1L proteins were conducted using DeepTMHMM, and the results showed that most SmCrRLK1Ls had one SP and TMH, which further demonstrated the membrane localization.

### 2.6. SmCrRLK1L Gene Promoter Analysis

**Figure 5:**
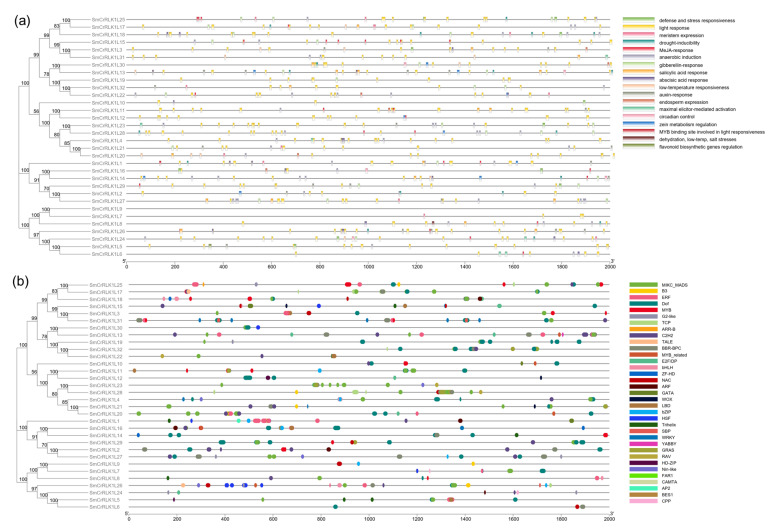
Promotor analysis of *SmCrRLK1Ls*. (**a**) The predicted cis-acting elements were distributed in *SmCrRLK1L* promotors. (**b**) The predicted transcription factor binding sites were distributed in *SmCrRLK1L* promotors.

To better study the putative functions of eggplant CrRLK1Ls, *SmCrRLK1L* gene promoters were extracted from the SGN database and analyzed using PlantCARE and PlantTFDB. The online PlantCARE tool was selected to predict the cis-acting elements. TBtools was used to visualize the results. As a result, 624 cis-acting elements were acquired, which could be divided into 18 featured categories, mainly related to defense, stress, light, drought, auxin, Methyl jasmonate (MeJA), gibberellin, abscisic acid (ABA), salicylic acid (SA), and low temperature responsiveness, suggesting that *SmCrRLK1L* may participate in hormone response, as well as the stress and defense responses (Dataset S5, Figure 5). Meanwhile, the transcription factor binding sites of *SmCrRLK1Ls* were analyzed using PlantTFDB. As shown in Figure 5 and Dataset S6, a total of 930 binding sites were determined in the *SmCrRLK1L* promoters, which belonged to 35 types of transcription factor, including MIKC_MADS, ERF, Dof, MYB, NAC, WRKY, and so on. The above results indicate that *SmCrRLK1L* may be widely involved in plant growth, development, and the host–pathogen interaction response.

### 2.7. SmCrRLK1L Gene Duplication Events

**Figure 6:**
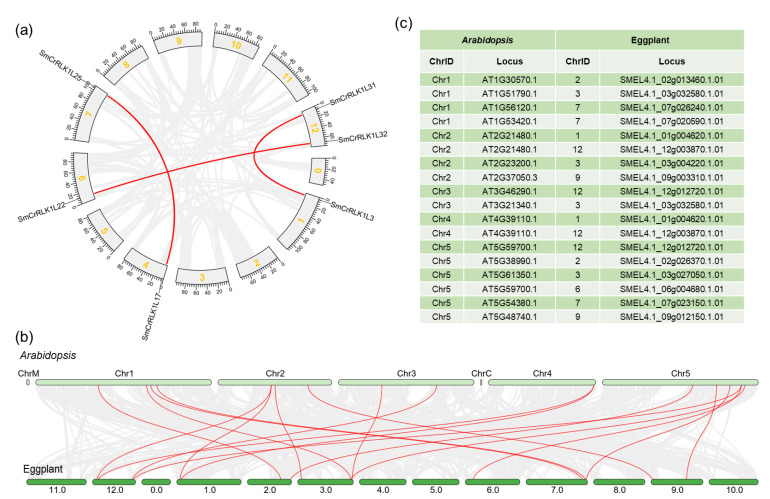
Collinearity of *SmCrRLK1Ls*. The collinearity relationships are marked with the red line. (**a**) The collinearity relationships of *SmCrRLK1Ls* within eggplant. Unit: Mb. (**b**,**c**) The collinearity relationships of *SmCrRLK1Ls* between eggplant and *Arabidopsis*.

To better understand the duplication events of the *SmCrRLK1L* genes, their collinearity was analyzed using One Step MCScanX from TBtools. As shown in Figure 6a, three pairs of *SmCrRLK1L* genes had collinearity relationships: *SmCrRLK1L3* with *SmCrRLK1L31, SmCrRLK1L17* with *SmCrRLK1L25*, and *SmCrRLK1L22* with *SmCrRLK1L32*. The result showed that these genes had intraspecific duplication events. Moreover, the collinearity of these genes between eggplant and *Arabidopsis* was also analyzed. As a result, 18 pairs of collinearity genes were obtained (Figure 6b,c), suggesting that these genes might have interspecific duplication events during evolution.

### 2.8. Expression Analysis of SmCrRLK1L Genes in Response to Botrytis cinerea Infection

Gray mold is caused by the fungus *Botrytis cinerea*, resulting in annual economic losses ranging from USD 10 to 100 billion worldwide. It ranks among the top ten fungal diseases affecting plants [51]. *Botrytis cinerea* is a necrotrophic fungus capable of infecting over 1400 plant species, including important economic crops such as tomato, strawberry, grapes, and eggplant [52]. Previous reports have indicated that the tomato CrRLK1L family member, SlFERL (*Solanum lycopersicum* FERONIA-like), plays a crucial role in the fruit of tomato’s resistance response to *Botrytis cinerea* [53]. To investigate the role of eggplant CrRLK1Ls in *Botrytis cinerea* infections, we utilized a transcriptomic analysis to study the *SmCrRLK1L* family transcription levels after exposure to *Botrytis cinerea*. The results revealed that the majority of genes exhibited a response to *Botrytis cinerea* infection. Specifically, *SmCrRLK1L1*, *5*, *6*, *15*, and *19* displayed strong upregulation, while *SmCrRLK1L2*, *11*, *12*, *17*, *18*, *22*, *26*, *27*, *29*, and *30* showed notable downregulation. These findings suggest that these genes may be involved in the interaction between eggplant fruit and *Botrytis cinerea*.

## 3. Discussion

CrRLK1Ls were identified in various plant species [48], while this family has not been identified in eggplant. There have been reports of CrRLK1Ls in various plant species, including 17 CrRLK1Ls in *Arabidopsis* [38], 16 in rice [39], 74 in apples [40], 62 in strawberries [41], 48 in tobacco [46], 17 in potato [47], and 24 in tomato [48]. In this study, our results showed that there were 32 SmCrRLK1Ls in eggplant, and each of them possessed the typical domains of CrRLK1Ls. When compared to other species in which the quantity of proteins in this family has been identified, eggplants have a greater number than *Arabidopsis* and rice, but fewer than apple and strawberry.

The phylogenetic analysis of eggplant and *Arabidopsis* CrRLK1Ls revealed that the SmCrRLK1Ls could be divided into three groups, with some having homologs in *Arabidopsis*. Various CrRLK1L members in *Arabidopsis* have been named and functionally characterized, such as ANJEA (ANJ), ANXUR1/2 (ANX1/2), CADMUS1/2 (CAD1/2), CURVY1 (CVY1), ERULUS (ERU), FERONIA (FER), HERCULES1/2 (HERK1/2), MODES1/2/3/4 (MDS1/2/3/4), BUDDHA’S PAPER SEAL1/2 (BUPS1/2), and THESEUS1 (THE1) [2].

Combining this with a collinearity analysis, we obtained potential homologous protein information for eggplant. For instance, SMEL4.1_02g013460.1.01 (SmCrRLK1L10) was clustered with AT1G30570.1 (AtHERK2), and their coding genes were predicted to form the collinear pairs, suggesting that SmCrRLK1L10 might be a homologous protein of AtHERK2. Similarly, SMEL4.1_12g012720.1.01 (SmCrRLK1L32) showed collinearity with AT3G46290.1 (AtHERK1), which was known to be involved in brassinosteroid (BR) response in *Arabidopsis* [33]. SmCrRLK1L10 and 32 may also participate in this hormone response. Additionally, SMEL4.1_01g004620.1.01 (SmCrRLK1L3) and SMEL4.1_12g003870.1.01 (SmCrRLK1L31) were identified as being collinear with AT4G39110.1 (AtBUPS1) and AT2G21480.1 (AtBUPS2). Meanwhile, these four proteins were clustered together in the phylogenetic analysis, indicating that SmCrRLK1L3 and 31 might be the homologs of AtBUPS1 and 2. AtBUPS1 and 2 were reported to be involved in pollen tube growth and the reactive oxygen species (ROS)-related stress response [12,34,54]. Furthermore, SMEL4.1_02g026370.1.01 (SmCrRLK1L11) was found to be clustered with AT5G38990.1 (AtMDS1), with a further collinearity analysis showing that these two genes were collinear, suggesting that SmCrRLK1L11 was a potential homolog of AtMDS1, which is involved in the metal ion stress response [35]. In addition, SMEL4.1_07g023150.1.01 (SmCrRLK1L25) was clustered with AT5G54380.1 (AtTHE1), indicating that SmCrRLK1L25 might be a homolog of AtTHE1, known to be involved in cell growth [32,33]. Future studies could explore whether SmCrRLK1L25 has a regulatory function in cell growth. The phylogenetic and collinearity analyses further revealed close relationships between SMEL4.1_03g027050.1.01 (SmCrRLK1L15) and AT5G61350.1 (AtCAP1/AtERU). AtERU/AtCAP1 mutants displayed a short root hair phenotype in *Arabidopsis* [8], suggesting that SmCrRLK1L15 mutants might have a similar phenotype. Additionally, our results also showed that SMEL4.1_03g004220.1.01 (SmCrRLK1L13) had collinear relationships with AT2G23200.1 (AtCAD2) and SMEL4.1_06g004680.1.01 (SmCrRLK1L22), with SMEL4.1_12g012720.1.01 (SmCrRLK1L32) showing a collinear relationship with AT5G59700.1 (AtANJ). In summary, one copy of SmCAD2 (SmCrRLK1L13), SmCAP1/ERU (SmCrRLK1L15), SmHERK1 (SmCrRLK1L32), SmHERK2 (SmCrRLK1L10), SmMDS1 (SmCrRLK1L11), and SmTHE1 (SlCrRLK1L25), as well as two copies of SmBUPS1/2 (SmCrRLK1L3, 31) and SmANJ (SmCrRLK1L22, 32), were identified in eggplant. These homologous proteins may share similar biological functions and warrant further functional elucidation in the future.

The analysis of the cis-acting elements and transcription factor binding sites in the SmCrRLK1Ls suggests their potential involvement in defense, stress, and hormone responsiveness. This aligns with previous findings for other plant species. Existing reports indicate that CrRLK1Ls play crucial roles in various aspects including plant development, fertility, environmental responses, and immunity in species such as *Arabidopsis*, tomato, rice, apple, strawberry, soybean, etc. [2]. For instance, in tomato, SlFERL (*Solanum lycopersicum* FERONIA-Like) interacts with SlSAMS1 (*Solanum lycopersicum* S-Adenosylmethionine Synthetase1) to modulate ethylene synthesis to regulate fruit ripening, and apple MdFERL (*Malus domestica* FERONIA-like) and strawberry FvMRLK (*Fragaria vesca* Malectin-like domain-containing Receptor-Like Kinase) are also involved in fruit ripening [21,41,55]. In rice, the expression of some *OsCrRLK1Ls* are controlled by circadian rhythms or drought, indicating that these genes are involved in circadian regulation or the drought stress response [39]. Furthermore, *Oryza sativa* FERONIA-like receptor1 (OsFLR1) and OsFLR2 have been identified as indispensable components responsible for maintaining plant architecture, reproduction, and seed yield [56,57]. Moreover, rice Ruptured Pollen tube (RUPO) play a pivotal role in regulating the growth and integrity of the pollen tube [58]. Glycine max lesion mimic mutant1 (GmLMM1) in soybean govern the pattern-triggered immunity (PTI) and cell death procedures, mounting defense against bacterial and oomycete pathogens’ invasion [59]. PbrCrRLK1L3 and PbrCrRLK1L26 in pears (*Pyrus bretchneideri*) are actively involved in the intricate processes of pollen tube rupture and growth [45]. The potato *StCrRLK1Ls* promoter region have a variety of cis-regulatory elements in response to plant hormones, defense and stress, and *StCrRLK1Ls’* expression has been altered after *Phytophthora infestans* infection, suggesting that they may play an important role in the response of potato to pathogenic fungi, and can be screened as candidate genes for further resistance research and functional analysis [47]. Upon *Pseudomonas syringae* infection, *Nicotiana benthamiana NbCrRLK1Ls* have displayed significant changes in expression, indicating that these genes may be involved in the response to *Pseudomonas syringae* [60]. Additionally, CqFER in *Chenopodium quinoa*, GmCrRLK1L20 in soybean, and NtCrRLK1L47 in tobacco have been documented to partake in the modulation of salt stress responses [42,46,61].

**Figure 7:**
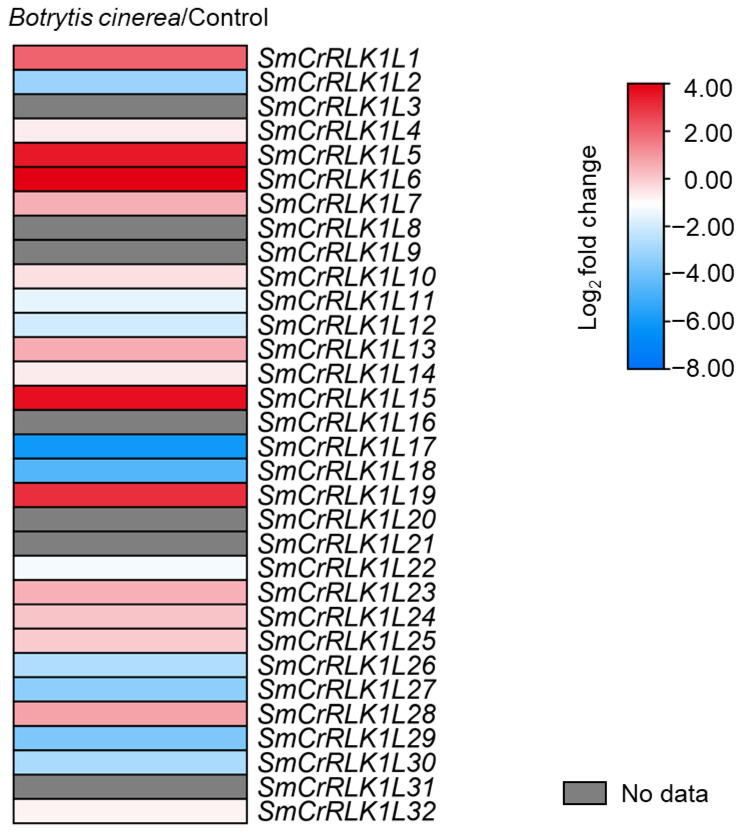
Expression analysis of CrRLK1Ls in eggplant upon *Botrytis cinerea* infection. Heatmap of *SmCrRLK1Ls’* expression after *Botrytis cinerea* infection. The data were acquired from our own transcriptome experiment.

Our integrating analysis of promoter elements indicated that SmCrRLK1Ls may also be involved in the above biological processes (Figure 5). A subsequent transcriptome analysis revealed that the expression of 15 *SmCrRLK1L* genes in eggplant fruits was induced with a *Botrytis cinerea* infection (Figure 7), suggesting that these genes may be involved in regulating eggplant’s resistance response to *Botrytis cinerea*, which is consistent with the promoter analysis results.

Previous research has reported that two or more genes within 200 kb on one chromosome is defined as a gene cluster [62]. Based on this criterion, the analysis revealed that *SmCrRLK1L5*, *6*, *7*, *8*, and *9* constitute one cluster on chromosome 2. Additionally, *SmCrRLK1L11* and *12* form a separate cluster on chromosome 2, while *SmCrRLK1L20* and *21* create a cluster on chromosome 6 (Figure 2, Dataset S2). It is conceivable that genes within a cluster might participate in the regulation of the same biological pathways, an aspect worth investigating in future studies.

## 4. Materials and Methods

### 4.1. SmCrRLK1L Members, Physicochemical Property Identification

The eggplant genome sequence was extracted from the Sol Genomics Network (SGN, https://solgenomics.net/) (accessed on 5 October 2022). *Catharanthus roseus*, rice, and *Arabidopsis* CrRLK1L sequences were downloaded from NCBI (https://www.ncbi.nlm.nih.gov/)(accessed on 5 October 2022), EnsemblPlants (http://plants.ensembl.org/index.html) (accessed on 5 October 2022) and TAIR (https://www.arabidopsis.org/)(accessed on 5 October 2022), respectively. Two HMM profiles, PK-Tyr-Ser-Thr (PF07714) and Malectin-like (PF12819), were served as entries to search for the SmCrRLK1L candidates (sequence and domain scores, E-value <0.05) by using TBtools [63]. The original data are shown in Dataset S1. VENNY (https://bioinfogp.cnb.csic.es/tools/venny/index.html) (accessed on 5 October 2022) was used to illustrate the Venn figures. ExPASy-ProtParam (https://www.expasy.org/resources/protparam) (accessed on 8 October 2022) was used to determine the predicted physicochemical properties of eggplant CrRLK1Ls.

### 4.2. Phylogenetic Analysis

Whole protein sequences of *Catharanthus roseus*, rice, *Arabidopsis*, and eggplant CrRLK1Ls were aligned using ClustalW. The neighbor-joining phylogenetic tree was constructed using MEGA 11 [64] with the following parameters: Poisson model, pairwise deletion, and 1000 bootstrap replicates.

### 4.3. Gene Location and Collinearity Analysis

The methods used in this study were as follows: All the location information of the eggplant genes were downloaded from the SGN (https://solgenomics.net/ftp/genomes/Solanum_melongena_V4.1/) (accessed on 5 October 2022); then, the information on the *CrRLK1L* genes was extracted and illustrated using TBtools. As for the collinearity analysis, eggplant and *Arabidopsis* genome data were obtained from TAIR and SGN. Then, the data were submitted to TBtools to determine the collinearity of the *CrRLK1L* genes. After the collinearity information was obtained, a visualization was conducted using Dual Synteny Plot from TBtools.

### 4.4. Protein Domain and Gene Structure Analysis

The eggplant CrRLK1L protein sequences were submitted to Pfam Scan (https://www.ebi.ac.uk/Tools/pfa/pfamscan/) (accessed on 7 October 2022) [65] to analyze the protein domain (E-value < 1 × 10^−10^). As for the transmembrane helix and signal peptide, we chose the DeepTMHMM (https://dtu.biolib.com/DeepTMHMM) (accessed on 11 October 2022) webtool to predict the detailed region information. The protein domain data are shown in Dataset S3. For the gene structure analysis, the *CrRLK1L* gene annotation file of eggplant was obtained from the SGN database and visualized using TBtools (Visualize Gene Structure tool, Chengjie Chen, Guangzhou, China).

### 4.5. Subcellular Localization Prediction

The whole CrRLK1L amino acid sequences of eggplant were submitted to the CELLO (http://cello.life.nctu.edu.tw/) (accessed on 9 October 2022) webtools and the most possible subcellular localization was determined using scores [66]. The original data are shown in Dataset S4.

### 4.6. Protein Conserved Motifs Analysis

The MEME webtool (https://meme-suite.org/meme/tools/meme) (accessed on 7 October 2022) was used to predict the conserved motifs of the eggplant CrRLK1L proteins. The protein sequences were submitted to MEME, 10 classic motifs were acquired, and the result was then visualized using TBtools.

### 4.7. Promoter Analysis

The 2000 bp upstream region of the *SmCrRLK1Ls’* start codon was obtained from the eggplant genome and considered as a predicted promotor. Then, the sequences were submitted to PlantCARE (http://bioinformatics.psb.ugent.be/webtools/plantcare/html/) (accessed on 11 October 2022) to identify the cis-acting elements, and PlantTFDB (http://planttfdb.gao-lab.org/index.php) (accessed on 11 October 2022) to identify the transcription factor binding sites, respectively. The detailed information is listed in Datasets S5 and S6 and was visualized using TBtools.

### 4.8. Transcriptomic Analysis

The eggplant (*Solanum melongena* L. var. *esculentum*) fruits were wounded with a sterilized scalpel, creating an incision 2 mm wide and 5 mm deep at the equator, prior to inoculation. Each wound site was then inoculated with 5 μL of a *Botrytis cinerea* spore suspension containing 2 × 10^5^ spores per mL. The total RNA of the eggplant was extracted from the infection part of the fruit and then delivered to Biomarker Technologies (Qingdao, China) for RNA-seq analysis. The detailed expression information is listed in Dataset S7.

## 5. Conclusions

In conclusion, this study utilized bioinformatic analyses to first identify and analyze 32 members of the CrRLK1L family in eggplant. The findings unveiled homologs of this family with well-known *Arabidopsis* CrRLK1Ls. Furthermore, an expression pattern analysis revealed that these genes may be involved in the interaction between the eggplant fruit and *Botrytis cinerea*. These studies have laid a theoretical foundation for the detailed functional identification of members in this family, offering new insights into disease resistance research for eggplant.

## Figures and Tables

**Table 1:** List of the eggplant CrRLK1Ls identified in the SGN database.

Protein Name	SGN Locus	Protein Length	MW	Theoretical pI	Instability Index	Aliphatic Index	GRAVY
SmCrRLK1L1	SMEL4.1_00g002980.1.01	916	102,981.91	9.05	38.81	89.29	−0.244
SmCrRLK1L2	SMEL4.1_00g011070.1.01	904	100,649.88	5.78	40.63	84.55	−0.284
SmCrRLK1L3	SMEL4.1_01g004620.1.01	878	96,435.51	6.03	42.89	84.54	−0.164
SmCrRLK1L4	SMEL4.1_01g027970.1.01	850	94,767.29	6.48	44.05	80.29	−0.256
SmCrRLK1L5	SMEL4.1_02g011520.1.01	990	111,146.47	8.55	35.88	89.65	−0.252
SmCrRLK1L6	SMEL4.1_02g011530.1.01	780	86,927.27	8.05	27.96	88.64	−0.27
SmCrRLK1L7	SMEL4.1_02g011560.1.01	956	106,793.65	5.85	36.2	90.76	−0.232
SmCrRLK1L8	SMEL4.1_02g011630.1.01	443	49,500.78	9.02	37.46	90.52	−0.258
SmCrRLK1L9	SMEL4.1_02g011650.1.01	930	103,516.67	7.27	35.74	92.49	−0.151
SmCrRLK1L10	SMEL4.1_02g013460.1.01	868	96,482.78	5.66	40.89	81.12	−0.273
SmCrRLK1L11	SMEL4.1_02g026370.1.01	1026	114,710.11	5.84	40.63	81.01	−0.273
SmCrRLK1L12	SMEL4.1_02g026380.1.01	997	112,040.66	6.38	41.89	84.84	−0.259
SmCrRLK1L13	SMEL4.1_03g004220.1.01	827	92,723.16	5.58	40.19	86.09	−0.207
SmCrRLK1L14	SMEL4.1_03g014350.1.01	610	67,859.65	5.85	31.72	96.05	−0.06
SmCrRLK1L15	SMEL4.1_03g027050.1.01	850	93,804.86	6.03	38.84	86.24	−0.09
SmCrRLK1L16	SMEL4.1_03g032580.1.01	834	93,418.44	5.78	32.53	94.21	−0.14
SmCrRLK1L17	SMEL4.1_04g002370.1.01	840	91,907.43	5.27	37.89	94.46	0.025
SmCrRLK1L18	SMEL4.1_05g003720.1.01	820	90,378.18	6.53	37.67	89.37	−0.065
SmCrRLK1L19	SMEL4.1_05g004710.1.01	819	91,513.23	5.65	34.6	90.13	−0.086
SmCrRLK1L20	SMEL4.1_06g000710.1.01	866	95,410.66	6.42	38.33	80.25	−0.298
SmCrRLK1L21	SMEL4.1_06g000720.1.01	911	100,056.89	6.11	34.63	82.52	−0.266
SmCrRLK1L22	SMEL4.1_06g004680.1.01	829	90,489.55	5.8	36.94	85.11	−0.077
SmCrRLK1L23	SMEL4.1_07g012220.1.01	885	96,959.49	5.77	43.66	79.51	−0.209
SmCrRLK1L24	SMEL4.1_07g020590.1.01	983	108,300.31	8.38	30.1	94.31	−0.108
SmCrRLK1L25	SMEL4.1_07g023150.1.01	843	92,227.57	5.51	37.69	89.18	−0.078
SmCrRLK1L26	SMEL4.1_07g026240.1.01	1058	116,977.8	6.3	31.28	92.28	−0.139
SmCrRLK1L27	SMEL4.1_09g003310.1.01	928	102,821.64	5.61	35.6	90.15	−0.181
SmCrRLK1L28	SMEL4.1_09g010930.1.01	888	97,206.73	5.69	42.59	80.01	−0.207
SmCrRLK1L29	SMEL4.1_09g012150.1.01	1084	120,775.58	5.77	46.93	97.1	−0.09
SmCrRLK1L30	SMEL4.1_10g010500.1.01	812	90,741.61	6.99	32.04	86.51	−0.118
SmCrRLK1L31	SMEL4.1_12g003870.1.01	847	93,940.44	5.44	35.97	81.89	−0.188
SmCrRLK1L32	SMEL4.1_12g012720.1.01	837	91,980.58	6.39	35.59	82.75	−0.125

**Table 2:** Subcellular localization prediction of SmCrRLK1Ls.

Protein Name	Signal Peptide Number	TMhelix Number	Most Likely Location
SmCrRLK1L1	1	1	Plasma Membrane
SmCrRLK1L2	1	1	Plasma Membrane
SmCrRLK1L3	1	1	Plasma Membrane
SmCrRLK1L4	1	1	Plasma Membrane
SmCrRLK1L5	1	1	Plasma Membrane
SmCrRLK1L6	1	1	Mitochondrial
SmCrRLK1L7	1	1	Cytoplasmic
SmCrRLK1L8	0	1	Plasma Membrane
SmCrRLK1L9	0	1	Plasma Membrane
SmCrRLK1L10	1	1	Plasma Membrane
SmCrRLK1L11	1	1	Plasma Membrane
SmCrRLK1L12	1	1	Plasma Membrane
SmCrRLK1L13	1	1	Plasma Membrane
SmCrRLK1L14	0	1	Plasma Membrane
SmCrRLK1L15	1	1	Plasma Membrane
SmCrRLK1L16	1	1	Plasma Membrane
SmCrRLK1L17	1	1	Plasma Membrane
SmCrRLK1L18	0	1	Plasma Membrane
SmCrRLK1L19	1	1	Plasma Membrane
SmCrRLK1L20	1	1	Plasma Membrane
SmCrRLK1L21	1	1	Plasma Membrane
SmCrRLK1L22	1	1	Plasma Membrane
SmCrRLK1L23	1	1	Plasma Membrane
SmCrRLK1L24	1	1	Plasma Membrane
SmCrRLK1L25	1	1	Plasma Membrane
SmCrRLK1L26	0	1	Plasma Membrane
SmCrRLK1L27	1	1	Plasma Membrane
SmCrRLK1L28	1	1	Plasma Membrane
SmCrRLK1L29	1	1	Plasma Membrane
SmCrRLK1L30	1	1	Plasma Membrane
SmCrRLK1L31	1	1	Plasma Membrane
SmCrRLK1L32	1	1	Plasma Membrane

## Data Availability

Not applicable.

## Supplementary Materials

The following supporting information can be downloaded at https://www.mdpi.com/article/10.3390/plants12193379/s1, Figure S1: Identification of eggplant CrRLK1Ls in the SGN database; Dataset S1: Simple HMM Search results of SmCrRLK1Ls identification; Dataset S2: Chromosome location information of *SmCrRLK1Ls*; Dataset S3: Detailed protein domain information of SmCrRLK1Ls; Dataset S4: Detailed information of SmCrRLK1Ls subcellular localization prediction; Dataset S5: Detailed cis-acting elements information of *SmCrRLK1Ls* promotor; Dataset S6: Detailed transcription factor binding site information of *SmCrRLK1Ls* promotor; Dataset S7: The detailed expression data of *SmCrRLK1L* genes in the transcriptome.
