# Supplementary material for: Genome-Wide Identification and Analysis of Catharanthus roseus Receptor-like Kinase 1-like Proteins in Eggplant
Source: Plants (Basel). 2023 Sep 25;12(19):3379. doi: 10.3390/plants12193379 (PMC10574150; doi:10.3390/plants12193379)
Supplement: Supplementary file 1 [file plants-12-03379-s001.zip › Supplementary Figure.pdf]

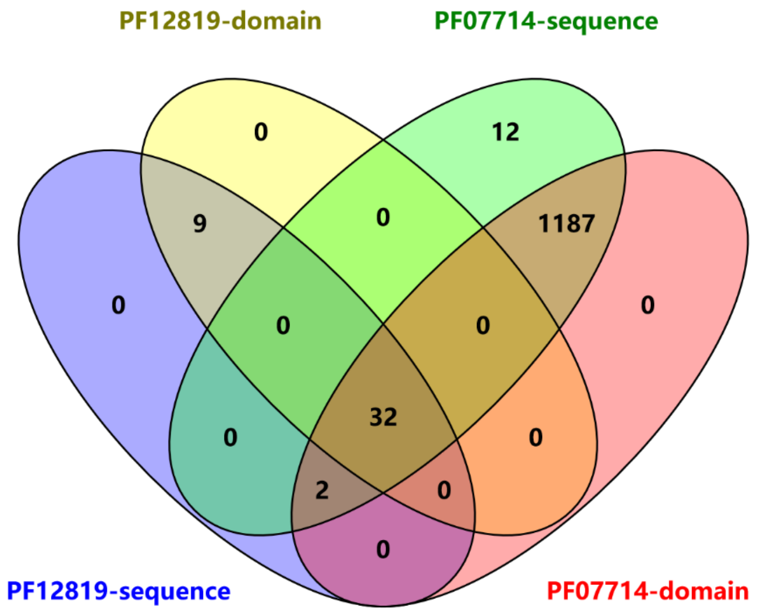

Figure S1. Identification of eggplant CrRLK1Ls in the SGN database. The predicted numbers are shown. PF12819: Malectin-like; PF07714: PK-Tyr-Ser-Thr. domain: The number of proteins that match HMM search domain score; sequence: The number of proteins that match HMM search sequence score. E-value < 0.05.
